# Racial Disparities in Food Insecurity for High- and Low-Income Households

**DOI:** 10.1001/jamahealthforum.2025.6935

**Published:** 2026-03-06

**Authors:** Cordelia Kwon, Yifan Liu, Deidra C. Crews, Boeun Kim, Elizabeth A. Stuart, Laura J. Samuel

**Affiliations:** 1 Graduate School of Arts and Sciences, Harvard University, Cambridge, Massachusetts; 2 School of Nursing, Johns Hopkins University, Baltimore, Maryland; 3 College of Nursing, University of Iowa, Iowa City; 4 Bloomberg School of Public Health, Johns Hopkins University, Baltimore, Maryland; 5 Statistical Editor for *JAMA Health Forum*

## Abstract

This cross-sectional study examines if intersectionality based on race and low-income status is related to food insecurity rates and the role that the Supplemental Nutrition Assistance Program plays.

## Introduction

Disparities in food insecurity based on race and income are well documented,^1^ as are the mitigating effects of the Supplemental Nutrition Assistance Program (SNAP)—a federal food aid program—on food insecurity.^2^ However, less evidence demonstrates if disparities in income explain racial differences in food insecurity or if other factors, such as structural racism, lead to disparities in racial groups at all income levels. In this study, we examine if intersectionality based on race and low-income status^3^ is related to food insecurity rates across 23 years and examine the role of SNAP.

## Methods

### Data Source

The Current Population Survey (CPS) annually recruits a nationally representative sample of community-dwelling individuals in the US aged 16 years and older through multistage probability-based sampling. We included CPS Food Security Supplement respondent households from 2000 through 2023 who had food insecurity measured using the validated 18-item US Department of Agriculture (USDA) food security module (n = 998 338); households endorsing 3 or more food-insecure conditions were classified as food insecure.^4^ Low-income status was defined as having a household income lower than 185% of the poverty threshold. Information about receiving SNAP benefits during the past 12 months was obtained. The household racial composition was classified as entirely entirely Asian or Pacific Islander, entirely Black, entirely Native American, White, or multiracial households.

### Statistical Analyses

This study was determined exempt from institutional review board review and written informed consent due to the use of publicly available, deidentified data. the study followed the Strengthening the Reporting of Observational Studies in Epidemiology (STROBE) reporting guidelines.

We estimated the national prevalence rates of food insecurity with 95% CIs for household food insecurity within subgroups based on race and income status. Group differences in proportions were compared using χ^2^ tests. Risk ratios among low-income households based on race and SNAP participation were estimated using a log-binomial regression. Asian or Pacific Islander were selected as the reference group. Household sampling weights were applied to all analyses to account for the complex survey design and nonresponse. Analyses were conducted using R statistical software (version 4.4.1; R Foundation). Analysis was conducted in 2025.

## Results

### Food Insecurity by Race and Income

Between 2000 and 2023, racial disparities in food insecurity persisted among both higher- and low-income households, with persistently higher rates for households that were entirely Black, Native American, or multiracial (Table 1). In addition, low-income households faced higher prevalence of food insecurity than higher-income households throughout the entire study period. For example, in 2023, 4.7% (95% CI, 3.1%-6.3%) of Asian and Pacific Islander higher-income households experienced food insecurity, compared with up to 16.6% (95% CI, 14.3%-18.8%) of Black high-income households (χ^2^ = 53.95, *P* < .001), 17.6% (95% CI, 13.0%-22.1%) of Asian and Pacific Islander low-income households, and 38.2% (95% CI, 35.0%-41.4%) of Black, low-income households (χ^2^ = 39.31, *P* < .001).

**Table 1.** Food Insecurity Proportions by Race and Income

Year	χ^2^ (95% CI)									
	Income >185% poverty level					Income <185% poverty level				
	Asian or Pacific Islander alone^a^	Black alone	Multiracial^b^	Native American alone	White alone	Asian or Pacific Islander alone^a^	Black alone	Multiracial^b^	Native American alone	White alone
2000	0.03 (0.02-0.05)	0.09 (0.08-0.11)	0.06 (0.04-0.08)	0.11 (0.05-0.17)	0.04 (0.04-0.04)	0.18 (0.14-0.22)	0.34 (0.31-0.36)	0.35 (0.29-0.41)	0.29 (0.21-0.37)	0.22 (0.21-0.23)
2005	0.03 (0.02-0.04)	0.13 (0.12-0.15)	0.06 (0.05-0.08)	0.10 (0.04-0.16)	0.05 (0.04-0.05)	0.14 (0.09-0.18)	0.35 (0.33-0.38)	0.37 (0.33-0.41)	0.35 (0.26-0.44)	0.24 (0.23-0.25)
2010	0.04 (0.03-0.06)	0.14 (0.12-0.16)	0.09 (0.07-0.11)	0.16 (0.07-0.25)	0.06 (0.06-0.07)	0.20 (0.16-0.24)	0.40 (0.37-0.42)	0.42 (0.37-0.46)	0.34 (0.25-0.43)	0.28 (0.28-0.29)
2015	0.02 (0.01-0.03)	0.12 (0.10-0.13)	0.08 (0.06-0.09)	0.16 (0.08-0.24)	0.05 (0.05-0.06)	0.16 (0.12-0.21)	0.36 (0.34-0.39)	0.35 (0.31-0.39)	0.37 (0.29-0.45)	0.28 (0.27-0.29)
2020	0.03 (0.02-0.04)	0.14 (0.12-0.16)	0.08 (0.07-0.10)	0.16 (0.09-0.23)	0.04 (0.04-0.05)	0.15 (0.11-0.20)	0.34 (0.31-0.37)	0.31 (0.26-0.35)	0.29 (0.18-0.39)	0.24 (0.22-0.25)
2023	0.05 (0.03-0.06)	0.17 (0.14-0.19)	0.11 (0.09-0.13)	0.17 (0.09-0.26)	0.06 (0.06-0.07)	0.18 (0.13-0.22)	0.38 (0.35-0.41)	0.37 (0.32-0.42)	0.41 (0.30-0.52)	0.26 (0.25-0.28)

^a^ Reference group.

^b^ Multiracial was defined as households entirely composed of multiracial individuals and/or households that contained individuals of multiple races.

### Food Insecurity by Race and SNAP Participation

Among low-income households, SNAP participants showed a less persistent pattern of racial disparities than nonparticipants (Table 2). In many years, the risk ratios comparing Asian or Pacific Islander with Black households were smaller among SNAP participants than non-SNAP participants, although not statistically significant.

**Table 2.** Food Insecurity Risk Ratios (RRs) Among Low-Income Households With and Without SNAP Compared With Asian and Pacific Islander Households

Year	RR											
	Black alone			Multiracial^a^			Native American alone			White alone		
	Not SNAP	SNAP	Interaction	Not SNAP	SNAP	Interaction	Not SNAP	SNAP	Interaction	Not SNAP	SNAP	Interaction
2000	1.65^b^	1.59	0.96	1.45	2.17	1.49	1.55	1.13	0.73	1.07	1.60	1.49
2005	2.24^b^	1.85	0.83	2.31^b^	2.16	0.93	2.31	1.78	0.77	1.55	1.74	1.12
2010	1.69^b^	1.73	1.02	1.77^b^	1.89^b^	1.07	1.27	1.69	1.33	1.22	1.59	1.31
2015	2.30^b^	1.31	0.57	1.88	1.50	0.80	2.41^b^	1.39	0.58	1.66	1.41	0.85
2020	1.81^b^	2.54	1.40	1.54	2.39	1.55	1.64	2.17	1.32	1.10	2.58	2.34
2023	1.93^b^	1.89	0.98	1.63	2.10^b^	1.29	1.54	2.44^b^	1.59	1.29	1.76	1.37

Abbreviation: SNAP, Supplemental Nutrition Assistance Program.

^a^ Multiracial was defined as households entirely composed of multiracial individuals and/or households that contained individuals of multiple races.

^b^ *P *< .001.

## Discussion

This cross-sectional study builds on prior evidence by documenting intersectional disparities persistent over 23 years despite multiple intervening policy and macroeconomic changes. As in a prior cross-sectional study,^5^ racial disparities were smaller among SNAP-participating households than nonparticipating households.

Further research is needed, particularly among Native American individuals, who consistently experienced high rates of food insecurity but have limited data, and multiracial households who represent a highly heterogenous group.

Nonetheless, these findings have important implications. Although the CPS Food Security Supplement survey was cancelled, these findings highlight the importance of national data to document disparities in food insecurity across US subgroups. Study findings that racial disparities existed among both higher- and low-income households indicate that income inequity is not the only mechanism through which racism impacts food insecurity. Cash and in-kind policies should consider differential impacts on racialized groups and address underlying discrimination.

## Notes

Supplement. Data Sharing Statement
