# Supplementary material for: Racial Disparities in Food Insecurity for High- and Low-Income Households
Source: JAMA Health Forum. 2026 Mar 6;7(3):e256935. doi: 10.1001/jamahealthforum.2025.6935 (PMC12966917; doi:10.1001/jamahealthforum.2025.6935)
Supplement: Supplement. — Data Sharing Statement [file jamahealthforum-e256935-s001.pdf]

Data Sharing Statement

Kwon. Racial Disparities in Food Insecurity for High- and Low-Income Households. *JAMA Health Forum*. Published March 06, 2026. doi:10.1001/jamahealthforum.2025.6935

Data
Data available: No

Additional Information
Explanation for why data not available: CPS data is publicaly available, so all data used is public. However, our cleaned and collated files used for this analysis will not separately be made public.
